# The Influence of Reducing Diets on Changes in Thyroid Parameters in Women Suffering from Obesity and Hashimoto’s Disease

**DOI:** 10.3390/nu13030862

**Published:** 2021-03-05

**Authors:** Lucyna Ostrowska, Dominika Gier, Beata Zyśk

**Affiliations:** 1 Department of Dietetics and Clinical Nutrition, Medical University of Bialystok, ul. Mieszka I 4 B, 15-054 Bialystok, Poland; lucyna.ostrowska@umb.edu.pl; 2 Faculty of Physiotherapy and Health Sciences, Gdansk College of Health, ul. Pelplińska 7, 80-335 Gdańsk, Poland; dominika.gier@gmail.com

**Keywords:** Hashimoto, obesity, elimination diet, thyroid gland, adults

## Abstract

Hashimoto’s disease is listed among the most common endocrine causes of obesity. As treatment of obesity in women with Hashimoto’s disease is frequently unsuccessful, the aim of this study was to evaluate the effectiveness of two different reducing diets and their influence on changes in thyroid parameters in female patients. A six-month observational/interventional study was performed on 100 women aged 18–65 years, previously diagnosed with Hashimoto’s disease and obesity and receiving L-thyroxine. The women were randomly assigned to the test group (group A, *n* = 50) following elimination/reducing diets, and the control group (group B, *n* = 50) following reducing diets with the same caloric content (without elimination). Anthropometric and thyroid parameters were evaluated at the beginning, after 3 months and after 6 months of treatment. In both groups a significant decrease in BMI and body fat percentage was achieved, but in test group A the decrease in BMI and body fat percentage was significantly greater than in control group B (*p* < 0.002 and *p* = 0.026, respectively). Serum TSH (thyroid stimulating hormon) levels decreased significantly more in group A than in group B (*p* < 0.001). Group A exhibited significantly greater increases in fT4 and fT3 levels than the control group (*p* < 0.001) as well as significantly greater decreases in the levels anti-TPO (thyroid peroxidase) (*p* < 0.001) and anti-TG (thyreoglobulin) antibodies (*p* = 0.048). The application of reducing diets with product elimination was found to be a more beneficial tool for changing anthropometric and thyroid parameters in women suffering from obesity and Hashimoto’s disease than classic reducing diets with the same energy values and macronutrient content.

## 1. Introduction

Hashimoto’s disease is also known as autoimmune thyroiditis or chronic lymphocytic thyroiditis. It is the most common type of thyroiditis and autoimmune endocrinopathy, constituting the most common non-iatrogenic cause of hypothyroidism [1]. The condition is more prevalent in the female population, and anti-TPO antibodies are more common in women (13.9%) than in men (2.8%) [2]. Weight gain is frequently the first symptom of hypothyroidism. The treatment of hypothyroidism (including autoimmune disorders) is based mainly on pharmacological treatment aimed at supplementing the deficiency of thyroid hormones and thus normalizing TSH levels. Research has shown that even when they achieve euthyroidism (normalization of thyroid hormones and TSH levels within laboratory norms), 82% of women treated still have excess body weight, and 35% of them suffer from obesity [3]. Currently, obesity is one of the most common diseases in Poland and in the world. According to the latest data the World Health Organization (WHO) date, the prevalence of obesity in the world has nearly tripled from 1975 to 2016 r. In 2016, over 1.9 billion (39%) adults were overweight and 650 million (13%) of them were obese. By distinguishing female gender, overweight was present in 40% of women and obesityin 15% of women [4]. In Poland in 2020, according to the report of the National Institute of Public Health—National Institute of Hygiene, the percentage of overweight people was 54%, and for those with obesity this was 10%. Taking into account the gender structure, overweight was reported in 46% of women and obesity in 8% of women [5]. The latest meta-analysis, covering 22 studies, showed that obesity is statistically significantly correlated with Hashimoto’s disease (*p* = 0.022) and high levels of anti-TPO antibodies (*p* = 0.001) [6]. This data also indicates that when hypothyroidism patients are treated with L-thyroxine, even after euthyroidism is reached, it is difficult to achieve effective weight reduction. This has prompted a search for more effective treatments for obesity in patients with Hashimoto’s disease. The aim of this study was, therefore, to evaluate the reducing/elimination diets based on calorie reduction and the obtained results of IgG_1-3_ hypersensitivity tests to individual food actigens, in terms of the effectiveness of weight reduction and the impact on thyroid parameters in patients suffering from obesity and Hashimoto’s disease. The use of elimination diets in food sensitivity is still controversial. Our aim was not to test the effectiveness of these diets in terms of the validity of their application (reduction of sensitivity, inflammation, autoimmunity, etc.), but to evaluate their effectiveness in patients suffering from two diseases that increase inflammation (obesity and Hashimoto’s disease). The problem of food sensitivity in the IgG_1-3_ class and its potential impact on body weight, inflammatory processes, and autoimmune diseases is currently of interest to scientists from around the world. In recent years several publications have confirmed the beneficial effects of elimination diets on metabolic and biochemical parameters in patients with excess body weight [7,8,9]. Both obesity and Hashimoto’s disease are inflammatory diseases.Both diseases are characterized by chronic low-grade inflammation and an overproduction of pro-inflammatory cytokines such as TNF-alpha and IL-6, so we are interested in elimination diets and the potential anti-inflammatory effects and clinical improvement associated with their application. There is experimental as well as clinical evidence that chronic inflammation can lead to increased extracellular water levels and water retention [10]. This effect can also be observed in patients with Hashimoto’s disease in the form of water accumulation in the glycosaminoglycans of connective tissue, which in turn causes subcutaneous edema [11]. In autoimmune patients, water retention in the body is statistically significantly greater than in healthy individuals (*p* < 0.05) [12].

## 2. Material and Methods

### 2.1. Subject

The interventional/observational study included 100 women aged 18–65 years with previously diagnosed Hashimoto’s disease and obesity. Hashimoto’s disease (AITD) was diagnosed by a specialist based on the ultrasound image characteristic of AITD and high levels of anti-thyroid antibodies. The study was approved by the Bioethics Committee of the Medical University of Białystok, no. R-I-002/187/2019. The women included in the study provided written consent and were supervised for six months by a dietitian and a physician. Upon their inclusion in the study, all of the women had BMI > 30 kg/m^2^ and received L-thyroxine, 200 mcg of 1-selenomethionine/day, and 30 mg of zinc gluconate/day, throughout the study period.

### 2.2. Study Protocol

The study included women diagnosed with Hashimoto’s disease visiting an Outpatient Clinic for obesity treatment. Data on the duration of Hashimoto’s disease and obesity as well as the current dose of L-thyroxine was collected based on medical history.

**Figure 1:**
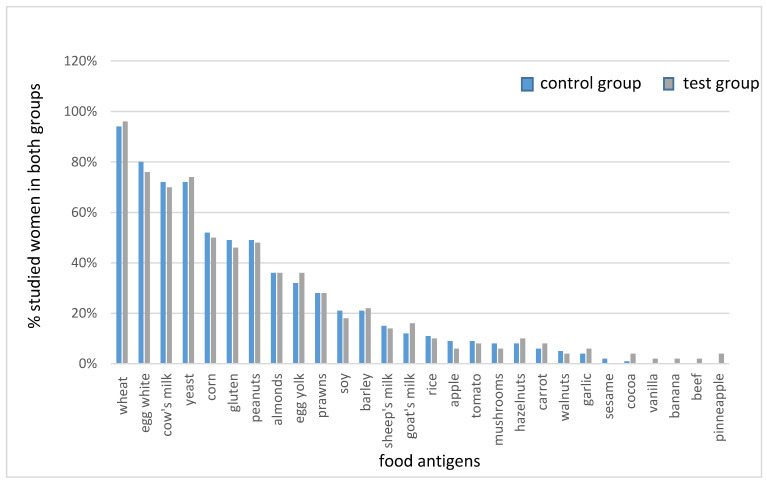
IgG_1-3_ food sensitivity in both studied groups.

All participants (*n* = 100) subsequently underwent laboratory tests for type III food sensitivity in the IgG_1-3_ class using the ELISA method. The tests were performed in an accredited medical laboratory. Figure 1 shows the results of tests for food sensitivity in both groups studied.

The women were randomly assigned to group A (the test group, *n* = 50) and group B (the control group, *n* = 50). The women from group A were then assigned to follow individually balanced elimination/reducing diets, in accordance with the previously performed food sensitivity tests (as shown in Figure 1). The elimination of foods from the menu was based on the individual results obtained.The remaining participants (group B) were assigned to follow individually balanced reducing diets (without elimination) for 6 months.

During the initial visit, all patients had their height and weight measured, and their body composition was analyzed using the bioimpedance method with a TANITA BC-420 body composition analyzer (T6360, Tanita Corporation, Tokyo, Japan). This examination was repeated during visits after 3 and 6 months. All women participating in the study also underwent laboratory tests to determine the following serum levels (at the initial visit, after 3 and 6 months): TSH, fT3, fT4, anti-TPO, and anti-TG. Blood samples were collected in an accredited medical laboratory, in the morning, on an empty stomach after a 12-h fast.

### 2.3. Diet Protocol

Both groups received diets in the range of 1400–1600 kcal/day (with a deficit of about 1000 kcal/day, depending on the resting metabolism and energy expenditure during the day). At the initial visit and every subsequent month, the women in both groups (A and B) received individually balanced menus composed according to their group assignment. In group B (control group), only caloric reduction was used. In the group A (test group), caloric reduction andindividual elimination of products were applied, in accordance with the obtained test results of participants of the study.All diets in both groups were designed by a qualified dietitian using the Aliant (Poland) diet calculator. Each diet had the same macronutrient content—25% protein, 30% fat, and 45% carbohydrate, and met the daily requirements for micro and macro elements for the given age group. Women in both groups also received 200 mcg of l-seleno-methionine/d and 30 mg of zinc gluconate/d throughout the study period.

**Figure 2:**
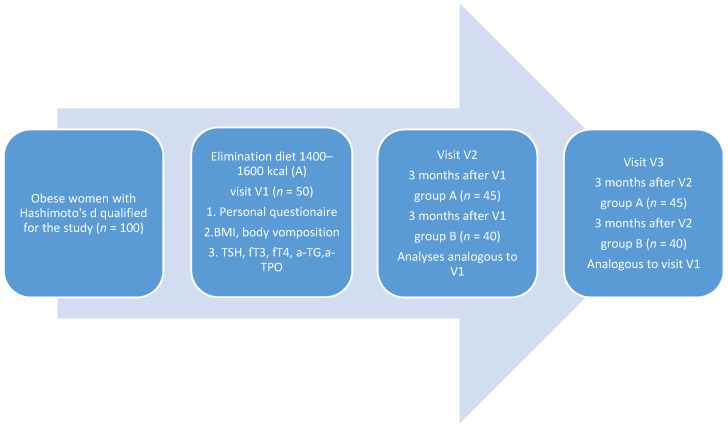
Experimental design. 100 obese women with Hashimoto’s disease were selected for the study. Stage 1 of the study (V1) included the completion of a personal questionnaire, assessment of body weight, height, and BMI, body composition analysis, biochemical tests: TSH, fT_3_, fT_4_, anti-TG, anti-TPO. Elimination diets of 1400–1600 kcal were recommended to fifty women (group A), while reducing diets of 1400–1600 kcal were recommended to the remaining 50 women—(group B). Stage 2 of the study (V2) began after 3 months and included analyses analogous to those conducted during V1. It included 45 women following elimination diets and 40 women following reducing diets. Next, 3 months after V2, the last, third stage of the study (V3) was performed. This included 45 women from the elimination group and 40 women from the reducing group. The tests performed at this stage were analogous to the previous stages.

The process of the study is shown in Figure 2.

### 2.4. Statistical Methods

In order to answer our research questions, we conducted statistical analyses using TIBCO’s Statistica 13.3 software (TibcoStatistica, California CA, USA). This was used to conduct analysis of basic descriptive statistics together with Shapiro-Wilk tests, mixed design multivariate analysis of variance, frequency analysis with the χ^2^ test, correlation analysis using Pearson’s r and Spearman’s ρ coefficients, and analysis with the independent samples t-test. The classic significance level of α = 0.05 (*p* < 0.05) was assumed. In the first step, the basic descriptive statistics of the studied quantitative variables were calculated together with the Shapiro-Wilk test to check the normality of the distribution of these variables. This test showed that the distribution of the following variables did not differ significantly from the normal distribution: body fat percentage (at the second and third visit), TSH level (at the second and third visit), and fT3 levels (at all three visits). A different situation was observed in terms of the age of the participants and other quantitative variables included in the study. In such cases, the value of skewness needs to be analyzed, and if its absolute value does not exceed 2, it can be assumed that the distribution is close to normal [13]. This was the case for all quantitative variables included in the study, except for anti-TPO antibody levels at the third visit. An outlier analysis (>+/−3SD) was performed for these variables, identifying two cases for anti-TPO antibody levels—both cases in the control group. After removing the outliers, an acceptable skewness value was obtained for all the listed variables. For changes in anti-TG antibody levels between the first and the third visit, a significant asymmetry of distribution in relation to the mean was observed—in this case outlier analysis was not performed, but non-parametric tests were used for testing hypotheses related to this variable. The remaining analyzed distributions were not significantly asymmetrical in relation to the mean. This is why for analyses including the remaining variables we used parametric tests, as long as their other assumptions were met. Analyses of correlations, with Pearson’s r and Spearman’s ρ coefficients, were performed between changes in medicine dose and changes in anti-thyroid antibody levels, between changes in BMI an thyroid hormone levels, and between changes in body fat content (%) and thyroid hormone levels. The correlation between body fat content (%) and TSH levels was also analyzed.

## 3. Results

The mean age of the women was 42.74 ± 10.51 in test group A, and 41.02 ± 11.96 in control group B; the groups were not statistically significantly different (*p* = 0.447). No statistically significant differences in the duration of obesity (*p* = 0.603) and Hashimoto’s disease (*p* = 0.159) were found between the two groups.

At the initial visit (V1), no statistically significant differences were found in the nutritional status of the studied women (group A—BMI 35.63 ± 4.06 kg/m^2^ vs. B—35.87 ± 5.59 kg/m^2^, *p* = 0.684); no differences were found also in their body fat percentage (group A—43.46 ± 3.77% vs. B—43.63 ± 4.05%; *p* = 0.830). There were also no statistically significant differences between the groups in terms of the serum levels of TSH (*p* = 0.908) and fT4 (*p* = 0.145). There was a statistically significant difference concerning the initial serum fT3 level (*p* = 0.037)—it was higher in control group B. There were no statistically significant differences between the groups in terms of the levels of anti-TPO (*p* = 0.283) and anti-TG (*p* = 0.829) antibodies.

Based on the results of IgG_1-3_ tests, the women from group A received individually adjusted elimination/reducing diets, while the women from group B received reducing diets (without ingredient elimination). The average caloric value of the elimination/reducing diets was 1516 ± 99.71 kcal/d in group A, and it was 1520 ± 98.97 kcal/d in group B (balanced reducing diets).

After the implemented nutritional intervention, test group A, following elimination/reducing diets, exhibited a statistically significant reduction in BMI values compared to the control group following reducing diets (*p* = 0.002). The results are presented in Table 1.

Before the beginning of the nutritional intervention, the groups did not differ significantly in terms of body fat content (*p* = 0.830). During the six-month follow-up, there was a decrease in body fat in both groups; in the test group by an average of 9.72%, and in the control group by an average of 7.19%. The reduction in body fat content was statistically significantly greater in the group following elimination/reducing diets (*p* = 0.026). The results are presented in Table 1.

Changes in serum levels of TSH, fT3, and fT4 were analyzed in both studied groups during the nutritional intervention. The results are presented in Table 2.

Post-hoc comparisons showed that a significant reduction in TSH levels occurs in both groups between the first and last visit, but these changes are more pronounced in group A; this difference was statistically significant in relation to group B (*p* < 0.001). During a six-month follow-up, group A exhibited a reduction of 48.03% in TSH levels compared to the baseline value, while group B had a reduction of only 24.54%. At the same time, a significant increase in fT4 levels was observed in both groups, and post-hoc comparison showed that the differences in fT4 levels between the first and last visit were significantly higher in group A (*p* < 0.001). Similar observations were made with regard to the behavior of fT3 levels. Post-hoc comparisons showed that group A exhibited significant increases in fT3 levels both between the first and second visits (*p* = 0.005) and between the second and third visits (*p* < 0.001), while in group B a significant increase was only visible between the first and the third visit (*p* < 0.001), but after six months of nutritional intervention the increase in this parameter level was significantly higher in group A (*p* < 0.001).

**Figure 3:**
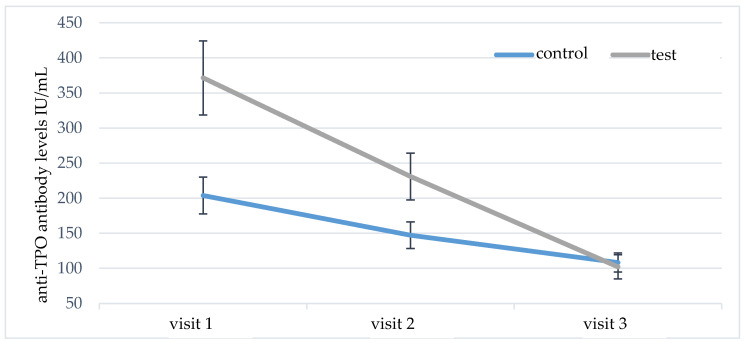
Comparison of test group A and control group B in terms of anti-TPO antibody levels during the six-month nutritional intervention.

The serum levels of anti-TPO and anti-TG antibodies were analyzed in a similar way. At the initial visit, significantly higher levels of anti-TPO antibodies were found in group A versus group B (*p* = 0.048). During the follow-up, there was a statistically significant decrease in anti-TPO antibodies in both studied groups (Table 2). However, post-hoc comparisons showed significantly higher reduction of anti-TPO antibodies in group A (*p* = 0.001); the results are shown in Figure 3.

**Figure 4:**
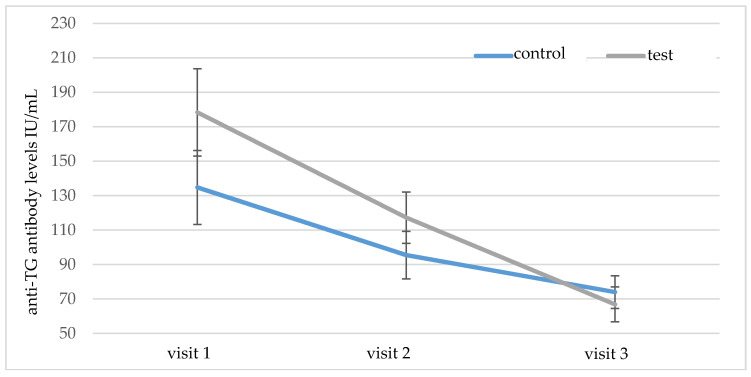
Comparison of test group A and control group B in terms of anti-TG antibody levels during the six-month nutritional intervention.

The mean initial serum level of anti-TG antibodies did not differ significantly between groups A and B (*p* = 0.829). During the six-month follow-up, the levels of these antibodies decreased significantly in both groups (Table 2), but post-hoc analysis showed that the reduction of anti-TG antibodies in group A was significantly higher (*p* = 0.048), as shown in Figure 4.

**Figure 5:**
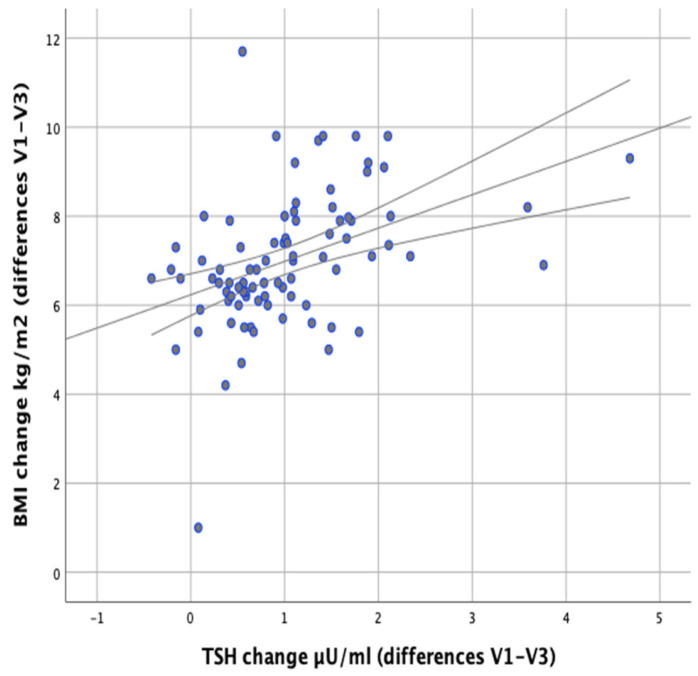
Correlations between differences in BMI and changes in serum TSH levels (between initial visit V1 and final visit V3).

**Figure 6:**
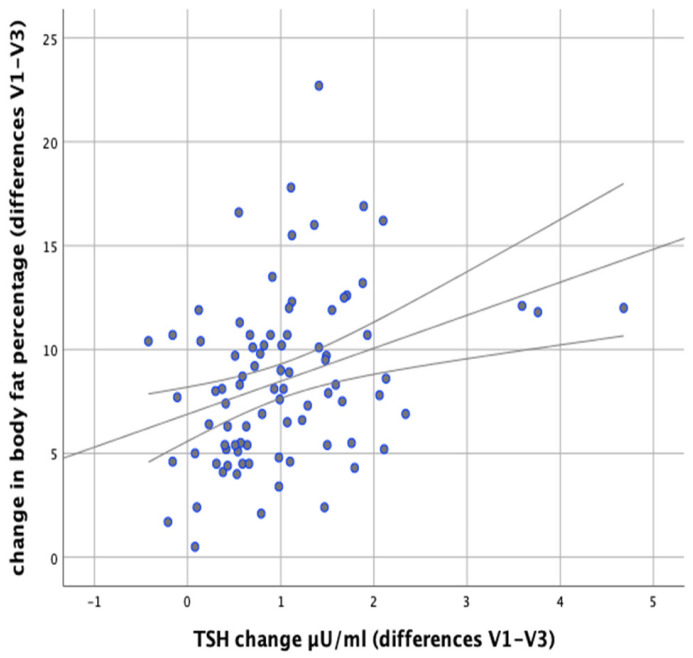
Correlations between changes in body fat content and differences in serum TSH levels (between initial visit V1 and final visit V3).

In our study we attempted to verify whether the observed changes in the levels of thyroid parameters correlate with changes in BMI and body fat content in the studied individuals. Significant positive correlations were found between the difference in TSH levels at the initial (V1) and final (V3) visits and the difference in BMI (V1–V3) (*r* = 0.500; *p* < 0.001) as well as between the difference in TSH levels and the difference in body fat content (*r* = 0.378; *p* < 0.001), which meant that BMI and body fat content reduction was accompanied by a decrease in TSH levels (Figure 5 and Figure 6).

**Figure 7:**
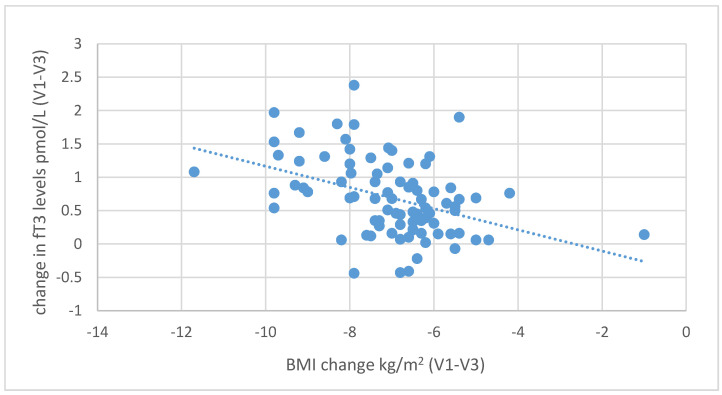
Correlations between differences in BMI and changes in serum fT3 levels (between initial visit V1 and final visit V3).

**Figure 8:**
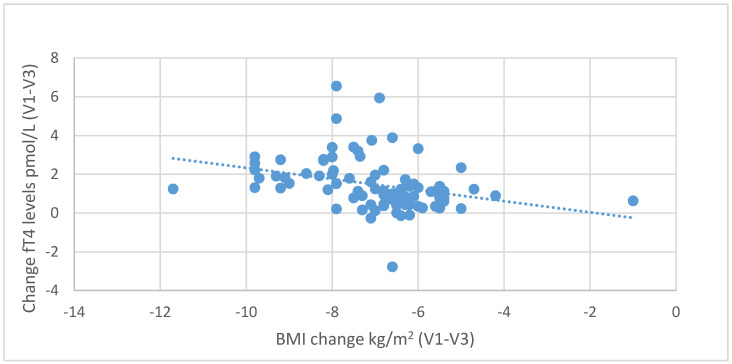
Correlations between differences in BMI and changes in serum fT4 levels (between initial visit V1 and final visit V3).

**Figure 9:**
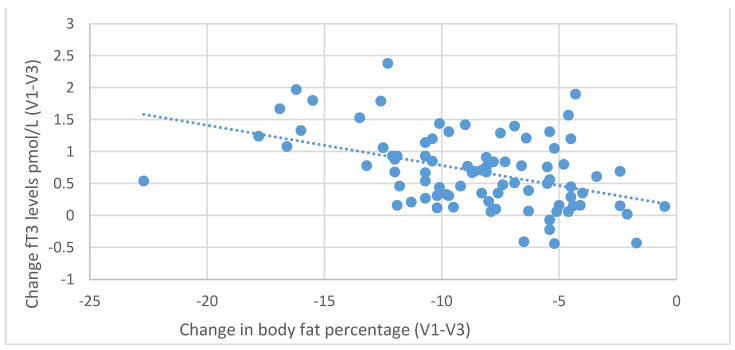
Correlations between differences in body fat content and changes in serum fT3 levels (between initial visit V1 and final visit V3).

**Figure 10:**
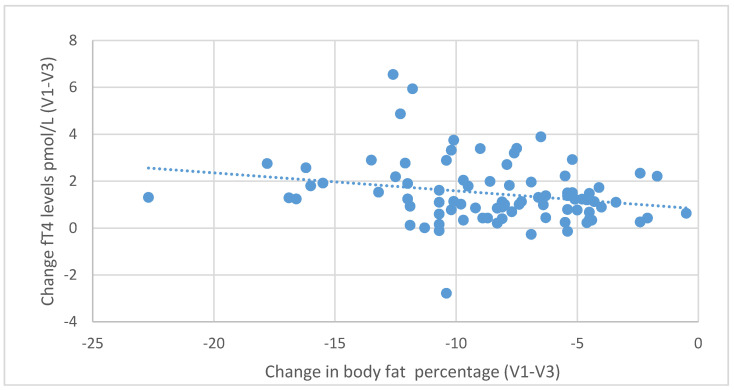
Correlations between differences in body fat content and changes in serum fT4 levels (between initial visit V1 and final visit V3).

During the six-month nutritional intervention, significant correlations were also observed between the reduction in BMI (difference between the values at the initial and final visit) and the increase in fT3 levels (*r* = 0.416; *p* < 0.001) and between the decrease in BMI and the increase in fT4 levels (r = 0.321; *p* = 0.003), as illustrated in Figure 7 and Figure 8. Negative correlations were also found between the decrease in body fat content and the increase in fT3 (*r* = 0.439, *p* < 0.001) and fT4 levels (*r* = 0.229, *p* = 0.035), as shown in Figure 9 and Figure 10.

**Figure 11:**
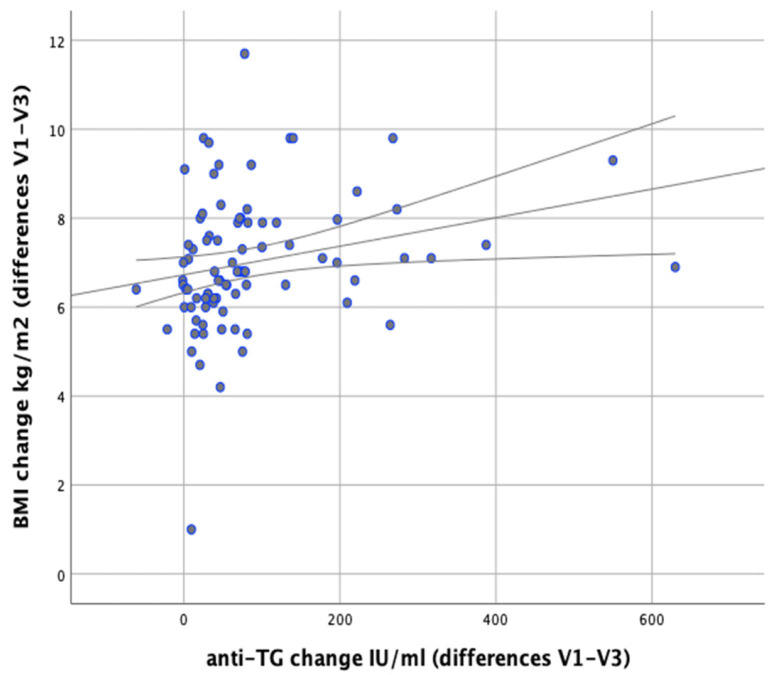
Correlations between differences in BMI and changes in serum anti-TG levels in the women studied (between initial visit V1 and final visit V3).

**Figure 12:**
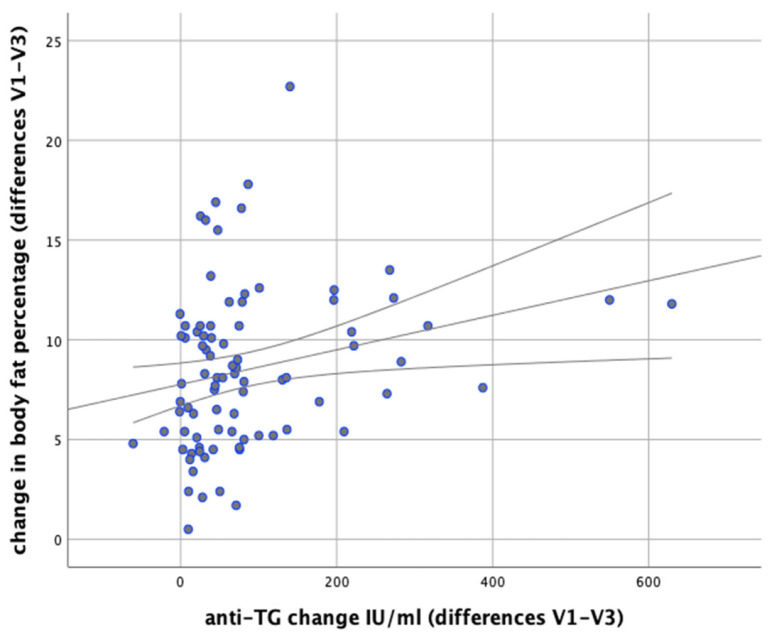
Correlations between changes in body fat content and changes in serum anti-TG levels in the women studied (between initial visit V1 and final visit V3).

In our analysis we attempted to verify whether any correlations exist between changes in body weight and changes in the levels of anti-thyroid antibodies during the implemented nutritional intervention. It was found that changes in body weight were significantly positively correlated with changes in the levels of anti-TG antibodies (*r* = 0.366; *p* = 0.001). Similarly, significant positive correlations were observed between changes in BMI and changes in anti-TG levels (*r* = 0.367; *p* < 0.001) and between changes in body fat content and changes in anti-TG levels (*p* = 0.003, *r* = 0.320), as illustrated in Figure 11 and Figure 12.

**Figure 13:**
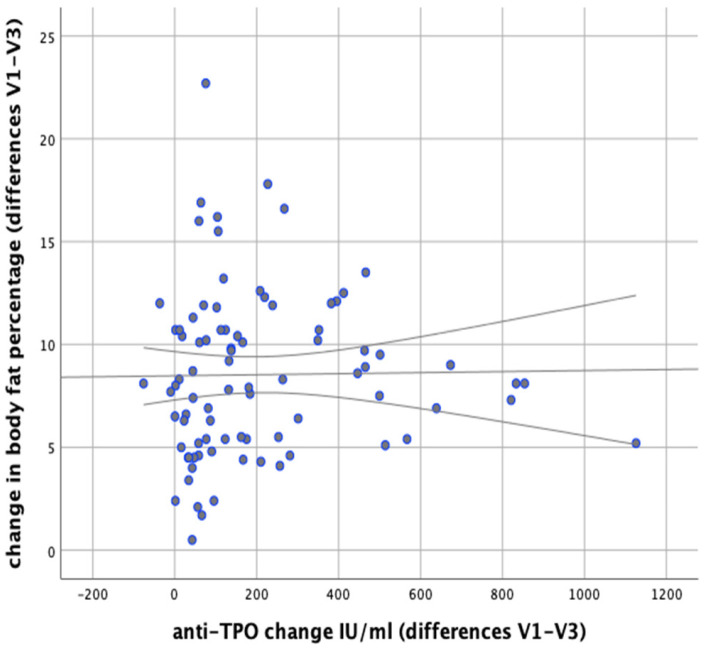
Correlations between changes in body fat content and changes in serum anti-TPO levels in the women studied (between initial visit V1 and final visit V3.

**Figure 14:**
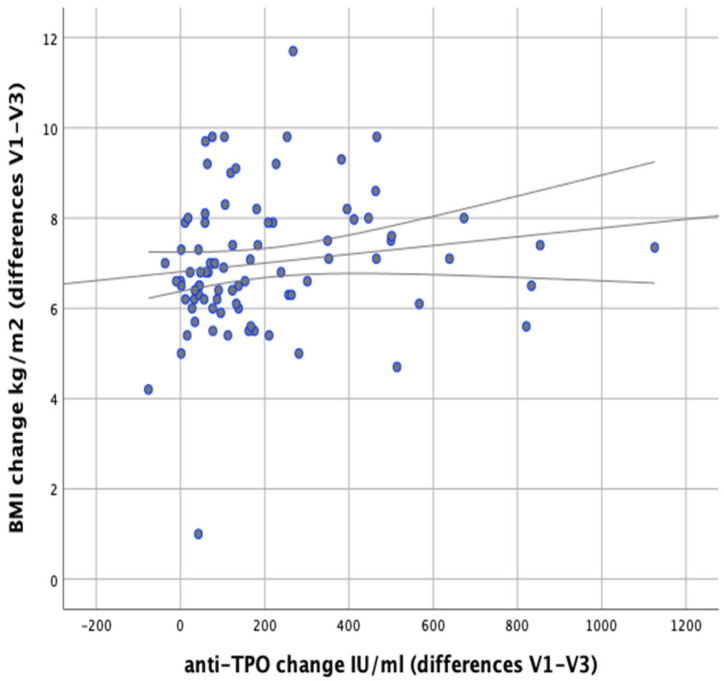
Correlations between differences in BMI and changes in serum anti-TPO levels in the women studied (between initial visit V1 and final visit V3).

There were no significant correlations observed between changes in anti-TPO levels and changes in body weight (*r* = 0.148; *p* = 0.177). There were no significant correlations revealed between changes in body fat content and anti-TPO levels either (*r* = 0.156; *p* = 0.154), as shown in Figure 13 However, a significant positive correlation was found between changes in BMI and changes in anti-TPO levels (*r* = 0.246; *p* = 0.023), as shown in Figure 14.

## 4. Discussion

Obesity is a huge global health problem, and the ever-growing number of people with excess body weight is a challenge for doctors and nutritionists. On the one hand, this situation requires specialists to take measures to prevent the growing obesity epidemic, and on the other it forces them to seek more effective forms of obesity treatment and weight reduction—in our case in women with Hashimoto’s disease. As shown by a meta-analysis covering 22 studies, obesity is statistically significantly associated with Hashimoto’s disease (*p* = 0.022) and high levels of anti-TPO (*p* = 0.001) [6]. Many studies indicate a strong relationship between Hashimoto’s hypothyroidism and excess body weight [14,15,16]. This is why the authors of many studies have posed the question of whether obesity in these patients is primary or secondary in nature. Omeljaniuket al. studied a group of patients diagnosed with Hashimoto’s disease; obesity was found in 28% of the participants [14]. In a study by Valea et al., among 100 women diagnosed with Hashimoto’s disease, obesity was found in 32% [15], while in a study by Szwajkosz et al., obesity was found in 39.6% of patients diagnosed with hypothyroidism [16].In a study by Koszowska et al., which included patients with autoimmune thyroiditis, it was shown that despite euthyreosis (normal levels of thyroid hormones and TSH), 82% of the patients had excess body weight, of whom 35% were obese [3], while 50% had an increased risk of metabolic disorders resulting from excessive amounts of body fat. Literature data indicates that despite L-thyroxine treatment, it is difficult to achieve body weight normalization in patients suffering from Hashimoto’s disease.

The primary method of treating obesity is changing eating habits and diet as well as increasing physical activity. It is only when non-pharmacological methods are ineffective that supportive treatment with the use of pharmacotherapy is implemented. Therefore, new nutritional models (diets) are still being sought to help reduce body fat and maintain reduced body weight. Assuming that the women in our study model had two diseases causing inflammation in the body, we decided to test whether we could achieve any clinical improvement in our patients using diets based on food sensitivity tests in the IgG_1-3_ class, which are still controversial.

IgG antibodies are the most strongly represented group of antibodies in the immune system and have as many as 4 subclasses. Subclasses 1-3 are the most abundant and have the ability to induce inflammation through complement components. In contrast, the least numerous subclass of IgG_4_ does not activate complement. IgG_1-3_ antibodies are involved in the type III hypersensitivity disorder described by Gell and Coombs over 50 years ago. Until now, this mechanism has been associated only with organ-nonspecific autoimmune diseases, and the pathology is based on the development of chronic inflammation caused by the production and local deposition of immune complexes. For some time, however, the immune mechanism involving IgG_1-3_ antibodies has also been associated with the possible development of hypersensitivity to food antigens. Underlying the hypothesis linking autoimmune diseases with type III hypersensitivity may be the unsealing of the intestinal barrier [17], without which a large amount of exogenous particles with antigenic properties could not appear in the tissues. It should also be mentioned that IgG antibodies have two Fab fragments and each is capable of being linked to one epitope (antigen-active fragment). In this way, immune complexes are formed, consisting of two antigen and antibody molecules. However, many antigens, including those derived from food proteins, have more than one identical antigen-active fragment (epitope), which gives a practically unlimited number of antigen-antibody combinations, and this opens the possibility of forming large, insoluble immune complexes. As a consequence of this phenomenon, complement activation can cause local inflammation, and with the constant deposition of immune complexes, chronic inflammation [18].

Effective weight reduction may lower inflammatory markers [19]. Studies by Wilders-Thrusing et al. [20] showed that obese individuals have significantly higher IgG_1-3_ antibody levels for various foods when compared to people with normal body weight. Additionally, these antibodies correlate with higher CRP (C-reactive protein) (*p* = 0.001), which may suggest their participation in ongoing low-intensity inflammation. Our research also confirmed the presence of positive IgG_1-3_ antibodies to food in women suffering from obesity and Hashimoto’s disease. A study by Carrocio et al. [21], including patients with non-celiac gluten sensitivity (NCGS), showed that the incidence of autoimmune disease in these patients was statistically significantly higher than in the control group without NCGS (*p* < 0.001). Hashimoto’s thyroiditis was the most frequently reported autoimmune disease associated with the presence of IgG antibodies to gliadin. Researchers also found that antinuclear antibodies (ANA)—used as a marker of autoimmune diseases—were present in 46% of NCGS patients compared with 2% of patients without NCGS. A study by Lambert and Vojdani [22], aimed at investigating the correlation between antibodies to food proteins and tissue antibodies, showed that patients with immunological reactivity to food proteins in the IgG_1-3_ class more frequently have tissue autoantibodies than patients in whom no food antibodies were identified. In the case of positive serum antibodies to gliadin, as many as 64% of patients developed antibodies to their own tissue antigens, compared with 35% of patients with tissue antibodies, but with no anti-gliadin antibodies detected. In a pilot study by Krysiak et al. [23], including women with autoimmune thyroiditis, it was found that a gluten-free diet may bring clinical benefits to patients suffering from Hashimoto’s disease; in the test group following a gluten-free diet, the level of anti-thyroid antibodies decreased statistically significantly (*p* < 0.001) when compared to the placebo group. Other researchers have also confirmed the potential benefits of using elimination diets to reduce inflammation in patients suffering from inflammatory diseases [24,25,26,27].

Therefore, in this study we attempted to use two reducing diets (a classic balanced reducing diet and a balanced reducing/elimination diet) to reduce body weight and improve thyroid parameters in women suffering from obesity and Hashimoto’s disease. The implemented nutritional interventions caused changes in anthropometric parameters in both groups participating in the study, but the results we obtained indicate a statistically significant difference in weight reduction in women following elimination diets as compared to standard reducing diets (*p* < 0.001). During the six-month nutritional intervention, the elimination diets enabled an average weight loss of 21.17 kg, and the reducing diets a weight loss of 17.03 kg (*p* < 0.001). In the control group, following classic reducing diets, the average reduction in body weight achieved in the first 12 weeks of the diet was 9.16 kg (0.76 kg/week), while the patients who followed elimination diets reduced their body weight during the first 12 weeks by 11.14 kg on average (0.92 kg/week); this result was statistically significant (*p* < 0.001). The results of the study in terms of BMI also show statistically significant differences after the elimination diets versus the reducing diets (*p* < 0.002). After the 6-month dietary intervention, body fat content was statistically significantly lower in the group of women on elimination diets than in the control group using classic reducing diets (*p* = 0.026). Similar results were obtained during six months of diet therapy by Onmus et al. [7] in a group of 82 patients with excess body weight and positive IgG antibodies to food proteins (with diets of 1600 kcal/day, similar to the ones used in our study). The differences in relation to the group using standard reducing diets were statistically significant in terms of weight reduction (*p* = 0.005), BMI (*p* = 0.001), and body fat content (*p* < 0.001). Similar results were obtained by Gubur S. [8] in her 3-month study including 50 patients with BMI > 30 kg/m^2^. A statistically significant reduction in body weight, BMI, and body fat was achieved (*p* < 0.05) with the use of an elimination diet A similar positive effect of elimination diets in terms of weight reduction was observed by Lewiset al. [9]. The study lasted 3 months and included 120 people. The effects of the diet associated with weight reduction were statistically significant (*p* < 0.01).

Since elimination diets have positive effects on weight reduction, we decided to check if and how they affect thyroid parameters and whether the obtained results depend only on weight reduction or also on the use of an elimination diet per se. The results of our study confirm that weight loss in women with Hashimoto’s disease has a positive effect on the reduction of serum TSH in both groups. Other researchers have also reached similar conclusions [28,29]. However, reduction of body weight with an elimination diet showed a stronger TSH lowering effect; the result was statistically significant in relation to the control group (*p* < 0.001). It was also noted that TSH levels at the last follow-up visit were significantly lower in patients following elimination diets than in patients from the group following reducing diets (*p* = 0.021), while before the nutritional intervention there was no statistically significant difference (*p* = 0.908). After six months of follow-up, we also observed a positive correlation between the difference in body fat content and TSH levels (*p* < 0.001). This confirms that the lower the body fat percentage observed in patients from both groups, the lower the TSH levels after the end of the nutritional intervention. After 6 months of nutritional intervention, the average TSH level in the test group following elimination diets was 1.45 μU/mL (reduction by 1.32 μU/mLon average), while in the control group following reducing diets it was 2.05 μU/mL (reduction by 0.75 µU/mLon average); the difference between the groups was statistically significant in favor of the test group (*p* < 0.001). de Moraes et al. also observed that patients with increased body fat content have higher TSH levels and reduced fT4 values [30]. Similarly, Rotondi et al. indicate that obesity may itself lead to changes in thyroid parameters [31]. The presence of TSH receptors in adipocytes [32] is associated with the influence of TSH on adipose tissue deposition under the skin and its participation in the regulation of pituitary TSH secretion and its involvement in the development of obesity. Thus, the lowering of TSH levels after a six-month nutritional intervention is a favorable phenomenon, especially in terms of effective obesity treatment. It is also important to remember that individuals suffering from obesity exhibit reduced expression of the TSHR and TRα1 genes in adipose tissue. This phenomenon is reversed as a consequence of the loss of excess body weight and reduction of body fat, resulting in the normalization of TSH and fT3 levels, which may additionally confirm the involvement of adipocytes in TSH secretion [33]. In our study, we also showed a positive correlation between BMI values and TSH levels (*p* < 0.001). Studies by Ruhla et al. also confirm the positive correlation of TSH with BMI, where patients with TSH >2.5 μU/mL had significantly higher BMI than those with lower serum TSH (30.47 ± 0.57 vs. 28.74 ± 0.18 kg/m2, *p* = 0.001) [34].

When it comes to thyroid hormones, we observed an increase in fT4 levels after a six-month nutritional intervention in both groups, but in the test group the increase was statistically significantly higher (*p* < 0.001). Additionally, a negative correlation was observed between serum fT4 and BMI (*p* = 0.003) and another correlation between decreased body fat content and increased fT4 levels (*p* = 0.035). Researchers reached similar conclusions when assessing thyroid function, BMI, and metabolic risk markers in a recently published cohort study involving almost 17,000 people [35]. They also observed a negative correlation between fT4 and BMI (*p* < 0.001). Similarly, after the application of our nutritional intervention in the treatment of obesity, fT3 levels increased statistically significantly more favorably in the group following elimination diets, when compared to the group following reducing diets only (*p* < 0.001). We also observed a negative correlation between fT3 levels and BMI (*p* < 0.001), and another correlation between increased fT3 levels and decreased body fat content (*p* < 0.001). Observing the levels of anti-thyroid antibodies provided an answer to the question of how these levels change in relation to body weight reduction. A significant decrease in a-TPO levels occurred in both groups, but in the test group it was statistically significantly higher (*p* < 0.001). In the group following elimination diets, we achieved a decrease in a-TPO by an average of 269.33 IU/mL, and in the group following reducing diets by an average of 95.59 IU/mL. The study showed that there is a positive correlation between a-TPO levels and BMI (*p* = 0.023); no such correlation was observed in the case of changes of a-TPO levels and decreased body fat percentage (*p* = 0.156). In light of the results obtained by Mazaheri et al. [36], effective reduction of the titer of antibodies to tissue peroxidase seems to be significant from the point of view of their potential negative effect on carbohydrate metabolism and prevention of insulin resistance in patients with Hashimoto’s disease. It should also be remembered that anti-TPO antibodies form immune complexes that activate the complement and T cells, thus contributing to an increase in the level of pro-inflammatory cytokines in patients with Hashimoto’s disease [37]. They play a key role in the development of hypothyroidism and, through the IgG_1_ class of antibodies, show a direct cytotoxic effect on the cells of the thyroid gland [37]. Moreover, increased titer of anti-TPO antibodies is associated with increased secretory function of both lymphocytes and monocytes, which in turn leads to the reactivation of B cells and production of anti-TPO antibodies [38]. Anti-TPOs are a good indicator of the thyroid autoimmunity process, as the higher the antibody titer, the more lymphocytic infiltrates in the thyroid gland and the faster the rate of damage to the thyroid gland [39,40]. Analysis of our study results showed that during the nutritional intervention and weight reduction, the levels of anti-TG antibodies were also reduced in both groups. Baseline antibody values did not differ significantly between the groups (*p* = 0.829), but the reduction in anti-TG antibody levels was statistically significantly greater in the group following elimination diets than in the group following reducing diets (*p* < 0.048). In the test group there was a decrease in anti-TG levels by an average of 111.45 IU/mL, while in the control group the average decrease was 60.8 IU/mL. We also observed a positive correlation between body fat percentage and anti-TG levels (*p* = 0.003), between BMI and anti-TG (*p* < 0.001), and between decreased body weight and anti-TG (*p* = 0.001).

## 5. Summary

Our research shows that improvement of thyroid parameters in patients suffering from obesity and Hashimoto’s disease is possible through effective weight reduction. Concurrently, an individually adjusted elimination diet may lead to better therapeutic results. The likely mechanism underlying the effectiveness of this diet may be associated with its anti-inflammatory effects. The details of the mechanisms associated with food IgG_1-3_ antibodies and their potential contribution to low-grade inflammation should be investigated in a larger population. Elimination diets were found to be a more effective tool in reducing body fat mass in women with Hashimoto’s disease than standard balanced reducing diets with the same energy value and main nutrient content.

## Figures and Tables

**Table 1:** Change in anthropometric parameters in both groups depending on the nutritional intervention used.

	Test Group A				Control Group B				(Test Group/Control Group)
Anthropometric Parameters	M	SD	Min	Max	M	SD	Min	Max	
body weight (kg) (visit 1)	98.26 *	±13.87	73	137	97.85 **	±16.73	76	164.6	*p* < 0.001
body weight (kg) (visit 2)	87.26	±13.60	64.1	125	89.32	±16.39	65.1	152.1	
body weight (kg) (visit 3)	77.77 *	±14.05	59.8	117.7	80.04 **	±17.14	58.4	146.9	
BMI kg/m2 (visit 1)	35.63 *	±4.06	30	48	35.87 **	±5.59	30.4	57	*p* < 0.002
BMI kg/m2 (visit 2)	31.62	±4.22	25.6	43.8	32.73	±5.53	26	52.6	
BMI kg/m2 (visit 3)	28.11 *	±4.69	21.4	41.2	29.16 **	±5.64	23.4	50.8	
body fat % (visit 1)	43.46 *	±3.77	37	52.9	43.63 **	±4.05	36.4	54.2	*p* = 0.026
body fat % (visit 2)	39.47	±5.00	27.8	52.1	40.56	±5.11	27	52.3	
body fat % (visit 3)	33.75 *	±6.84	22.1	51.2	36.32 **	±5.78	24.5	51.9	

M—mean, SD—standard deviation, Min—minimum value, Max—maximum value. * statistical significance between visits 1–3 in the test group (*p* < 0.001); ** statistical significance between visits 1–3 in the control group (*p* < 0.001).

**Table 2:** Change in thyroid parameters in both groups depending on the nutritional intervention used.

	Test Group A (Elimination/Reducing Diet)				Control Group B (Reducing Diet)				*p* (Test Group/Control Group)
Thyroid Parameters	M	SD	Min	Max	M	SD	Min	Max	
TSH (µU/mL) (visit 1)	2.77 *	±1.05	0.82	6.65	2.80 **	±1.22	0.23	6.54	*p* < 0.001
TSH (µU/mL) (visit 2)	2.00	±0.77	0.67	4.08	2.33	±0.78	0.71	4.23	
TSH (µU/mL) (visit 3)	1.45 *	±0.58	0.36	2.57	2.05 **	±0.68	0.65	3.41	
fT4 (pmol/L) (visit 1)	14.12 *	±1.58	11.80	18.92	14.61 **	±1.84	11.30	22.80	*p* < 0.001
fT4 (pmol/L) (visit 2)	15.00	±2.04	12.07	22.07	15.03	±1.44	12.78	20.76	
fT4 (pmol/L) (visit 3)	16.18 *	2.08	13.08	22.81	15.43 **	±1.42	13.22	20.02	
fT3 (pmol/L) (visit 1)	3.88 *	±0.67	2.41	5.15	4.22 **	±0.53	3.11	5.31	*p* < 0.001
fT3 (pmol/L) (visit 2)	4.18	±0.76	2.76	5.99	4.46	±0.54	3.12	5.79	
fT3 (pmol/L) (visit 3)	4.79 *	±0.73	2.94	6.74	4.66 **	±0.51	3.71	5.96	
aTPO(IU/mL) (visit 1)	371.49 *	±348.39	7.44	1567.00	203.80 **	±182.01	7.00	678.90	*p* < 0.001
aTPO(IU/mL) (visit 2)	230.80	±217.81	9.20	946.70	147.26	±132.16	11.00	458.10	
aTPO(IU/mL) (visit 3)	102.16 *	±116.02	5.46	441.30	108.26 **	±83.94	5.00	318.90	
aTG(IU/mL) (visit 1)	178.33 *	±166.21	8.00	638.90	134.78 **	±190.32	8.90		*p* = 0.048
aTG(IU/mL) (visit 2)	117.24	±98.89	8.54	408.60	95.48	±133.49	9.23	597.80	
aTG(IU/mL) (visit 3)	66.88 *	±67.56	3.42	276.60	73.98 **	±60.23	9.44	299.80	

M—mean, SD—standard deviation, Min—minimum value, Max—maximum value. * statistical significance between visits 1–3 in the test group (*p* < 0.001); ** statistical significance between visits 1–3 in the control group (*p* < 0.001).
